# Potential Role of Rainbow Trout Erythrocytes as Mediators in the Immune Response Induced by a DNA Vaccine in Fish

**DOI:** 10.3390/vaccines7030060

**Published:** 2019-07-03

**Authors:** Sara Puente-Marin, Ivan Nombela, Veronica Chico, Sergio Ciordia, Maria Carmen Mena, Luis Perez, Julio Coll, Maria del Mar Ortega-Villaizan

**Affiliations:** 1 Departamento de Bioquímica y Biología Molecular, Instituto de Investigación, Desarrollo e Innovación en Biotecnología Sanitaria de Elche (IDiBE) and Instituto de Biología Molecular y Celular (IBMC), Universidad Miguel Hernández (UMH), 03202 Elche, Spain; 2 Unidad de Proteómica, Centro Nacional de Biotecnología (CNB-CSIC), 28049 Madrid, Spain; 3 Departamento de Biotecnología, Instituto Nacional de Investigación y Tecnología Agraria y Alimentaria (INIA), Biotecnología, 28040 Madrid, Spain

**Keywords:** rainbow trout, erythrocytes, red blood cells, GVHSV, transcriptome, proteome, antigen presentation

## Abstract

In recent years, fish nucleated red blood cells (RBCs) have been implicated in the response against viral infections. We have demonstrated that rainbow trout RBCs can express the antigen encoded by a DNA vaccine against viral hemorrhagic septicemia virus (VHSV) and mount an immune response to the antigen in vitro. In this manuscript, we show, for the first time, the role of RBCs in the immune response triggered by DNA immunization of rainbow trout with glycoprotein G of VHSV (GVHSV). Transcriptomic and proteomic profiles of RBCs revealed genes and proteins involved in antigen processing and presentation of exogenous peptide antigen via MHC class I, the Fc receptor signaling pathway, the autophagy pathway, and the activation of the innate immune response, among others. On the other hand, GVHSV-transfected RBCs induce specific antibodies against VHSV in the serum of rainbow trout which shows that RBCs expressing a DNA vaccine are able to elicit a humoral response. These results open a new direction in the research of vaccination strategies for fish since rainbow trout RBCs actively participate in the innate and adaptive immune response in DNA vaccination. Based on our findings, we suggest the use of RBCs as target cells or carriers for the future design of novel vaccine strategies.

## 1. Introduction

Aquaculture is one of the fastest growing food production sectors [1] and the development of prophylactic measures against viral infections is one of the focal points in the sector. Among these measures, vaccination is one of the main strategies, and DNA vaccines, developed in recent years, are the most promising type of prophylaxis in aquaculture. DNA vaccination triggers effective protection [2] and stimulates cellular and humoral immune responses [3]. In the case of *Novirhabdovirus*, a viral genus responsible for high economic losses in aquaculture [4], only glycoprotein G has shown effectiveness as a DNA vaccine [5,6]. However, the extrapolation of effective DNA vaccines against a broad range of fish pathogens has been unsatisfactory. Currently, the mechanisms and cells involved in the protection triggered by DNA vaccines are still not fully understood and more research is necessary in order to improve the effectiveness of DNA vaccines. In this sense, fish nucleated red blood cells (RBCs) have emerged in the last decade as novel participants in the immune response of non-mammalian vertebrates [7,8].

RBCs have been involved in the fish immune response by expressing cytokines [8,9,10,11,12,13,14] and acting as immune cell mediators against viral pathogens [8]. Nucleated RBCs can carry and respond to a DNA vaccine encoding glycoprotein G of viral hemorrhagic septicemia virus (GVHSV) to modulate the expression of genes related to type 1 interferon (IFN1), antioxidant enzymes, and antigen presentation genes and provide protection to other cell types against VHSV infection and crosstalk with other cell lines in vitro [14].

Regarding RBCs, in recent decades, mammalian RBCs, which are not nucleated, have been proposed as possible drug and vaccine carriers [15,16,17,18] because of their capacity to induce effective immune responses comparable to traditional vaccination [15]. Additionally, DNA vaccines directed to antigen-presenting cells (APCs) have demonstrated improved humoral and cellular responses compared to non-targeted vaccines in mammalian models [19,20,21]. Considering that fish RBCs have been proposed to behave as atypical APCs [22], the strategy of targeting DNA vaccines or immunostimulants to RBCs represents a new approach in the field of fish prophylaxis.

Taking everything into account, including the fact that the role of nucleated RBCs in the immune response has gained interest recently, our aim was to explore the currently unknown role of rainbow trout RBCs in the context of in vivo DNA vaccination. In this study, we show for the first time the role of rainbow trout RBCs in the global host immune response to a DNA vaccine. Our results show that rainbow trout RBCs can modulate their transcriptome and proteome in response to a DNA vaccine encoding GVHSV. In addition, these RBCs can act as cell mediators of the immune response to activate antigen presentation, blood and head kidney immune cell signaling, and hematopoiesis. RBCs transfected in vitro with GVHSV can boost leukocyte proliferation, increasing the number of cells and leukocyte-specific markers. Moreover, reinfusion of autologous GVHSV-transfected RBCs in vitro induced VHSV-specific antibodies in vivo.

## 2. Material and Methods

### 2.1. Animals

Rainbow trout (*Oncorhynchus mykiss*) of approximately 7–10 cm (for transcriptomic and proteomic assays) and 20–25 cm (for RBC reinfusion assays) were obtained from a VHSV-free commercial farm (PISZOLLA S.L., CIMBALLA FISH FARM, Zaragoza, Spain). Fish were maintained at the University Miguel Hernandez (UMH) facilities at 14 °C and fed daily with a commercial diet (Skretting, Burgos, Spain). Prior to experiments, the fish were acclimatized to laboratory conditions for 2 weeks.

### 2.2. Plasmids

The plasmids pmTFP1 (Allele Biotechnology, ABP-FP-TCNCS), encoding the teal fluorescent protein 1 (mTFP1) [23], used as control (referred to as TFP1), and pmTFP1GVHSV, encoding mTFP1 fused to the C-terminus of the GVHSV (GenBank accession A10182.1) [24], (referred to as GVHSV), both with the promoter of human cytomegalovirus (CMV) and the termination signal of simian virus 40 (SV40) PolyA, were used for in vivo rainbow trout DNA vaccine injection and in vitro RBCs transfection assays. The efficacy of the DNA vaccine encoding the gene of GVHSV, with CMV promoter and SV40 termination signal, has been previously verified in vivo [25,26,27] and in vitro [14,24].

### 2.3. DNA Immunization

For transcriptomic and proteomic analyses, juvenile rainbow trout (7–10 cm) were anesthetized with tricaine (tricaine methanesulfonate, Sigma-Aldrich, Madrid, Spain) (40 mg/L) and injected intramuscularly (im) with 10 µg of TFP1 plasmid, or GVHSV plasmid in 50 µL of phosphate buffered saline (PBS) using insulin syringes (NIPRO, Bridgewater, NJ, USA). At 14 days post-injection (dpi), fish were sacrificed by overexposure to tricaine (0.3 g/L), and peripheral blood and head kidney were recovered. The sample collection time point was selected based on previous *gvhsv* gene transcripts expression monitorization in blood and head kidney (data not shown) and in the bibliography [3,28].

For the GVHSV-transfected RBC reinfusion assay, adult rainbow trout (20–25 cm) were anesthetized with 40 mg/L tricaine and reinfused intravenously (iv) with previously extracted autologous peripheral blood RBCs (PB-RBCs) (15 × 10^6^ cells) that were GVHSV-transfected in vitro as previously described [14]. For the in vitro transfection of RBCs, Ficoll-purified PB-RBCs were transfected by electroporation with 4 μg of GVHSV plasmid per 10^6^ cells using the Neon™ Transfection System (Life Technologies, Thermo Fisher Scientific, Inc., Waltham, MA, USA). Fish were immunized with im or iv injection of 4 μg GVHSV for immunization controls. At 30 dpi, blood was drawn from the caudal vein and left overnight at 4°C to separate the serum from the cell pellet.

### 2.4. Purification of Head Kidney and Peripheral Blood RBCs for Transcriptome Analysis, by Means of FACS Single-Cell Sorting

Head kidney and peripheral blood from individuals immunized with GVHSV or TFP1 were extracted at 14 dpi, and sampled in RPMI-1640 medium (Dutch modification) (Gibco, Thermo Fischer Scientific Inc., Carlsbad, CA, USA) supplemented with 10% fetal bovine serum (FBS) gamma irradiated (Cultek, Madrid, Spain), 1 mM pyruvate (Gibco), 2 mM L-glutamine (Gibco), 50 µg/mL gentamicin (Gibco), 2 µg/mL fungizone (Gibco), 100 U/mL penicillin (Sigma-Aldrich), and 100 μg/mL streptomycin (Sigma-Aldrich). Head kidney tissue was disaggregated with a Pasteur pipette and passed through a 40 μm Falcon nylon cell strainer (BD Biosciencies, Madrid, Spain) using a plunger of a 1 mL syringe. Then, cells from head kidney and peripheral blood were stained with 500 nM SYTO RNA Select (Molecular Probes, Thermo Fisher Scientific, Inc.) for 20 min at room temperature as recommended by the manufacturer. Head kidney RBCs (HK-RBCs) and peripheral blood RBCs (PB-RBCs) were FACS single-cell sorted using a BD FACSJazz™ cell sorter (BD Biosciences, Madrid, Spain) using SYTO RNA Select staining, which stains RNA and allows to sort populations based on cell RNA quantity. RBCs population for sorting was selected based on SYTO RNA select staining (FITC) and side scatter light (SSC), as indicated in Supplementary Figure S1, using the mask 1.0 drop single. Then, samples were visualized in the IN Cell Analyzer 6000 Cell Imaging system (GE Healthcare, Little Chalfont, UK) to verify a purity of ≥99.99% (Supplementary Figure S1).

### 2.5. Transcriptome Analysis of RBCs

Thirty-two individuals (16 for TFP1 injection and 16 for GVHSV injection) were immunized as described above. At 14 dpi, approximately 10^2^ HK-RBCs and 10^6^ PB-RBCs of each individual were sorted as described in the previous section.

**Figure 1:**
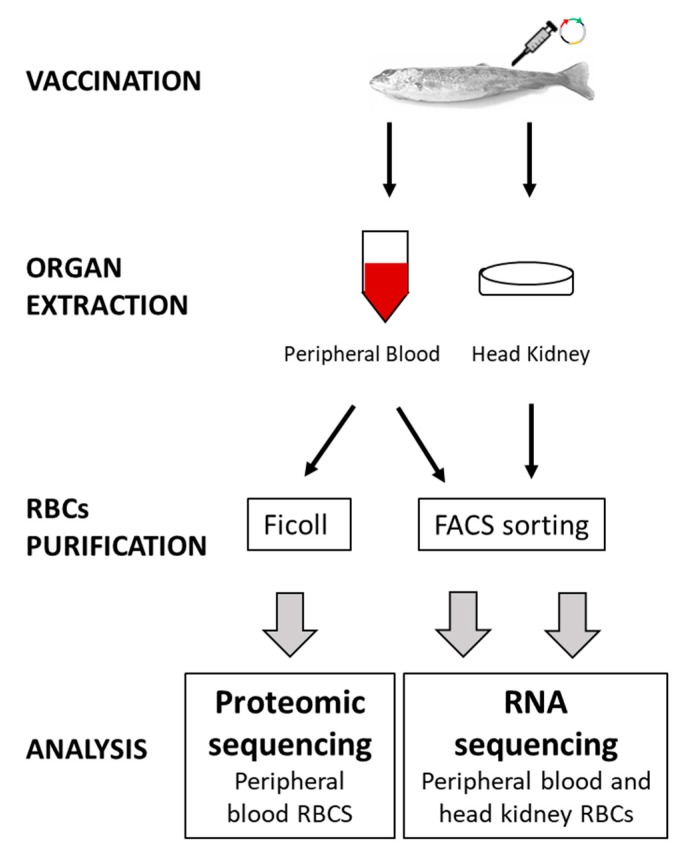
The general workflow of experimental steps from sample collection, 14 dpi, to data analysis.

Each sample was resuspended in lysis buffer (Clontech, Takara Bio, Mountain View, CA, USA) and RNase Inhibitor (Invitrogen, ThermoFisher Scientific Inc.) as indicated in Reference [14] and then grouped in 2 pools of 8 individuals for each condition (TFP1 or GVHSV) and tissue (HK-RBCs or PB-RBCs) (Figure 1). Samples were preserved at −80 °C until cDNA library construction. cDNA was directly produced from pooled lysed cells using the SMART-Seq v4 Ultra Low Input RNA Kit (Clontech, Takara Bio). RNA-Seq library preparation and sequencing were carried out by STABVida Lda (Caparica, Portugal) as previously described [29]. Sequence reads are available at SRA-NCBI, SRA-NCBI Accession SRP133501.

### 2.6. Proteome Analysis of RBCs

Thirty-two individuals (16 for TFP1 injection and 16 for GVHSV injection) were immunized as described above. Peripheral blood was extracted at 14 dpi, and PB-RBCs were purified by 2 density gradient centrifugations (1600 rpm, Ficoll 1.007; Lymphoprep, Reactiva, Sigma-Aldrich) as previously described [9]. The 99.9% purity of RBCs was estimated by optical microscopy (Supplementary Figure S1). Cells (10^7^ cells per individual) were pelletized by centrifugation (1600 rpm, 5 min), the supernatant was removed, and the cell pellet was washed 3 times with PBS. The pellet was then digested, cleaned-up/desalted, and pooled into 2 pools of 8 individuals for each condition (TFP1 or GVHSV) (Figure 1). Samples were subjected to liquid chromatography and mass spectrometry analysis (LC-MS) as previously described [14,29].

### 2.7. Pathway Enrichment Analysis

Pathway enrichment analysis of differentially expressed genes (DEGs) and differentially expressed proteins (DEPs) were performed using the Cytoscape version 3.6.1 [30] with ClueGO version 2.5.0 [31] and CluePedia version 1.5.0 [32] plugins. The Gene Ontology (GO) Biological Process and GO Immune System Process Databases were used with a *p*-value ≤ 0.05 and a *p*-value ≤ 0.5, respectively, and Kappa score of 0.4 as threshold values. The STRING v11 (http://string.embl.de/) [33] software was used to analyze protein-protein interaction (PPI) networks, with a medium confidence score threshold of 0.4. The *Homo sapiens* model organism was used for ClueGO and STRING analyses. Genes and proteins were identified by sequence homology with *Homo sapiens* using Blast2GO version 4.1.9 (Biobam, Valencia, Spain) [34] as previously described [29].

### 2.8. RNA Extraction, cDNA Synthesis, and RT-qPCR Gene Expression

RNA was isolated using E.Z.N.A. Total RNA Kit (Omega Bio-Tek, Inc., Norcross, GA, USA) following the manufacturer’s instructions together with DNAse (TURBO DNase, Ambion, Thermo Fisher Scientific, Inc.) and quantified with a NanoDrop Spectrophotometer (Nanodrop Technologies, Wilmington, DE, USA). cDNA synthesis and quantitative reverse transcription polymerase chain reaction (RT-qPCR) were performed using the ABI PRISM 7300 System (Applied Biosystems, Thermo Fisher Scientific, Inc.) as previously described [9]. Specific primers and probes are listed in Table 1. The gene encoding EF1α was used as an endogenous control.

### 2.9. Coculture of Transfected RBCs with White Blood Cells

Ficoll-purified RBCs from peripheral blood were transfected with TFP1 or GVHSV plasmid as indicated previously [14]. Transfected RBCs were cocultured with autologous Ficoll-purified white blood cells (WBCs) from peripheral blood on 96-well plates for 7 days at 14 °C at a ratio of 10^5^ RBCs/10^5^ WBCs per well. WBCs (cocultured with untransfected RBCs) treated with phytohemagglutinin-L (Sigma-Aldrich) (PHA-L) were used as a positive control of lymphocyte proliferation because PHA-L is a well-known lymphocyte proliferation compound [46,47,48]. After 7 days of coculture, WBCs proliferation was evaluated via cell counting and RT-qPCR of lymphocyte cell markers. For cell counting, cells were stained with Hoechst (Sigma-Aldrich) and counted using the IN Cell Analyzer 6000 workstation 3.7.2 software (GE Healthcare, Little Chalfont, UK). Cell counting by means of cell imager systems has been previously performed for cell proliferation assays [49]. WBC proliferation was calculated using the following formula: ((number of cell nuclei in WBCs and treated RBCs − number of cell nuclei in untreated RBCs) ÷ (the number of cell nuclei in WBCs and control RBCs − number of cell nuclei in untreated RBCs)) × 100. WBCs cocultured with TFP1-transfected RBCs were used as control. Untreated and no cocultured RBCs were used as negative control. For RT-qPCR, samples were stored at −80 °C in lysis buffer until RNA extraction and RT-qPCR analysis.

### 2.10. Enzyme-Linked Immunosorbent Assay (ELISA)

Serum was collected from immunized or reinfused individuals at 30 dpi as indicated above. After centrifugation for 15 min at 3500 rpm, the serum was stored at −20 °C until used. Negative serum was collected from unimmunized individuals and positive serum was collected from VHSV-challenged survivors. VHSV-specific IgM antibodies were measured by ELISA as previously described [26] with minor modifications. Briefly, 96-well plates were coated with concentrated VHSV (0.5 µg/well in PBS) and dried overnight at 37 °C. Then, immunized fish serum dilutions ranging from 1/30–1/240 diluted in PBS with 0.05% Tween (Sigma-Aldrich) and 0.5% bovine serum albumin (BSA) (Sigma-Aldrich) were applied to each well and incubated for 2 hours at room temperature (RT). Plates were washed 3 times with PBS-0.05% Tween. Then, plates were incubated with a primary monoclonal antibody against trout IgM (1G7) [50] diluted 1/200 in PBS-0.05% Tween for 90 min at RT. Plates were washed 3 times with PBS-0.05% Tween and then incubated with rabbit anti-mouse conjugated with peroxidase (RAM-Po) (Sigma-Aldrich) diluted 1/500 in PBS-0.05% Tween for 1 hour at RT. Finally, plates were washed 3 times with PBS-0.05% Tween and incubated with the 1-Step ultra TMB-ELISA (Thermo Fisher Scientific, Inc.) as a substrate for the peroxidase reaction for 20 to 30 min at RT. Absorbance was measured at 450 nm in an Eon microplate reader (BioTek, Winooski, VT, USA).

### 2.11. Statistical Analysis

The GraphPad Prism 6 software (GraphPad Software Inc., San Diego, CA, USA)(www.graphpad.com) was used for statistical analysis.

### 2.12. Ethics Statement

Experimental protocols and methods relating to experimental animals were reviewed and approved by the Animal Welfare Body and the Research Ethics Committee at the University Miguel Hernandez (approval number 2014.205.E.OEP; 2016.221.E.OEP) and by the competent authority of the Regional Ministry of Presidency and Agriculture, Fisheries, Food and Water supply (approval number 2014/VSC/PEA/00205). All methods were carried out in accordance with the Spanish Royal Decree RD 53/2013 and EU Directive 2010/63/EU for the protection of animals used for research experimentation and other scientific purposes.

## 3. Results

### 3.1. RNA Sequencing of HK-RBC from GVHSV Immunized Rainbow Trout

Transcriptome profiling of HK-RBCs that were FACS single-cell sorted from GVHSV-immunized individuals (Figure 1) identified 479 DEGs (false discovery rate [FDR] < 0.05); 287 were upregulated and 192 were downregulated when compared to HK-RBCs from TFP1-injected individuals (Supplementary Table S1). *gvhsv* gene transcripts were detected, but not significantly (FDR > 0.05), in HK-RBCs from GVHSV-immunized individuals.

**Figure 2:**
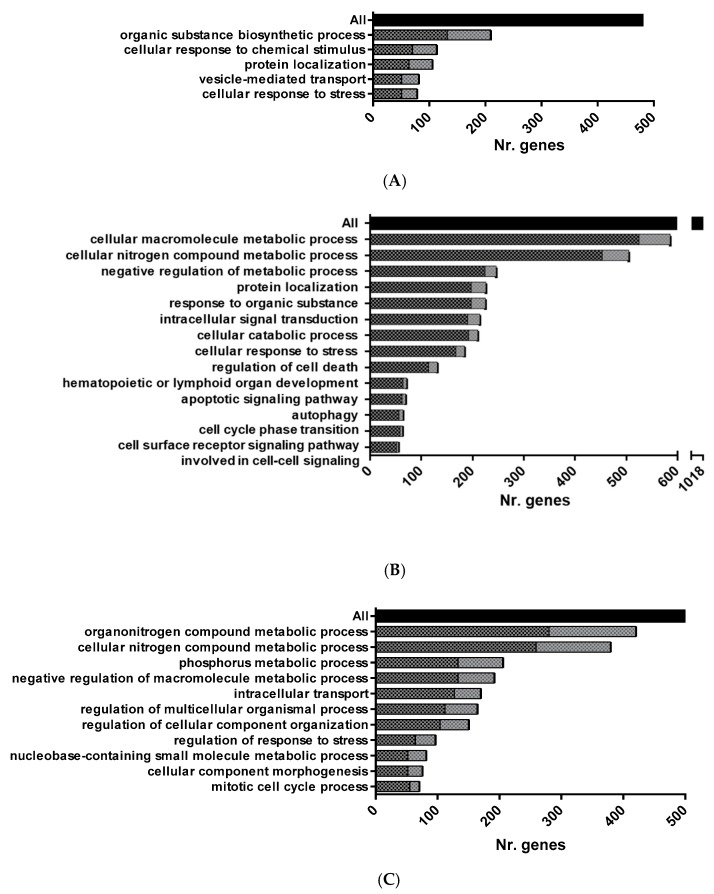
Overrepresented functional pathways in RBCs from GVHSV-immunized fish. Main overrepresented GO Biological Process Database terms were identified by the Cytoscape ClueGO. (**A**) Overrepresented pathways in HK-RBC transcriptome profile. (**B**) Overrepresented pathways in PB-RBC transcriptome profile. (**C**) Overrepresented pathways in PB-RBC proteome profile. Black squares represent upregulated genes or proteins, and gray squares represent downregulated genes or proteins identified in each GO term. The black bar represents the total number of genes or proteins with FDR < 0.05 and FC *p*-value < 0.05. All GO terms overrepresented were statistically significant with *p*-value < 0.05.

Functional pathway enrichment analysis of DEGs in HK-RBCs from GVHSV-immunized individuals using the GO Biological Process Database revealed overrepresentation of the following categories: organic substance biosynthetic process, cellular response to chemical stimulus, protein localization, vesicle-mediated transport, and cellular response to stress (Figure 2A) (Supplementary Tables S2 and S3). Among the identified cellular response pathways, we were particularly interested in chemokines C-X-C motif chemokine receptor 4 (*cxcr4*) (log_2_ fold change (FC) = 13.01), C—C motif chemokine receptor 9 (*ccr9*) (log_2_ FC = 13.07), C—C motif chemokine ligand 25 (*ccl25*) (log_2_ FC = 8.63), and C—C motif chemokine ligand 13 (*ccl13*) (log_2_ FC = 8.97), all of which are involved in leukocyte chemotaxis.

**Figure 3:**
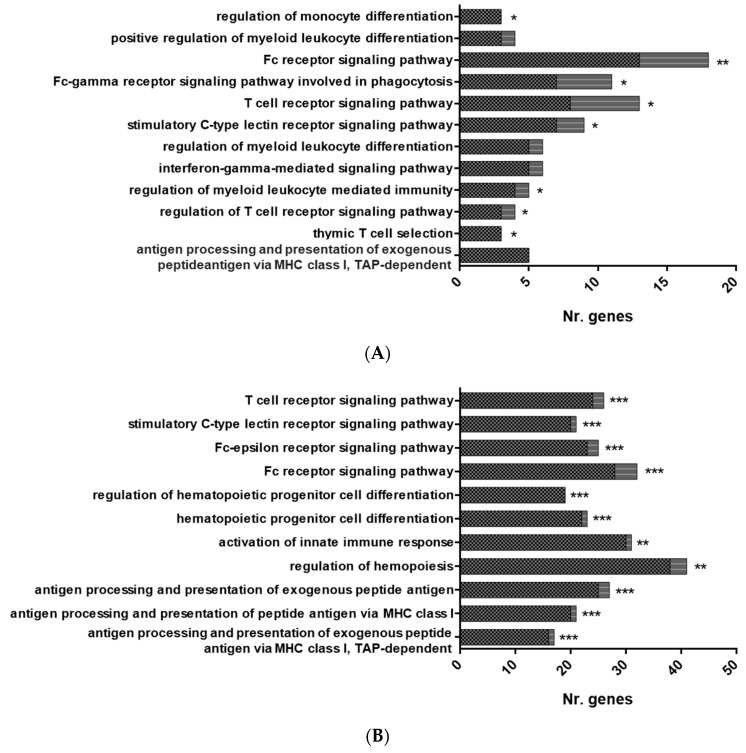

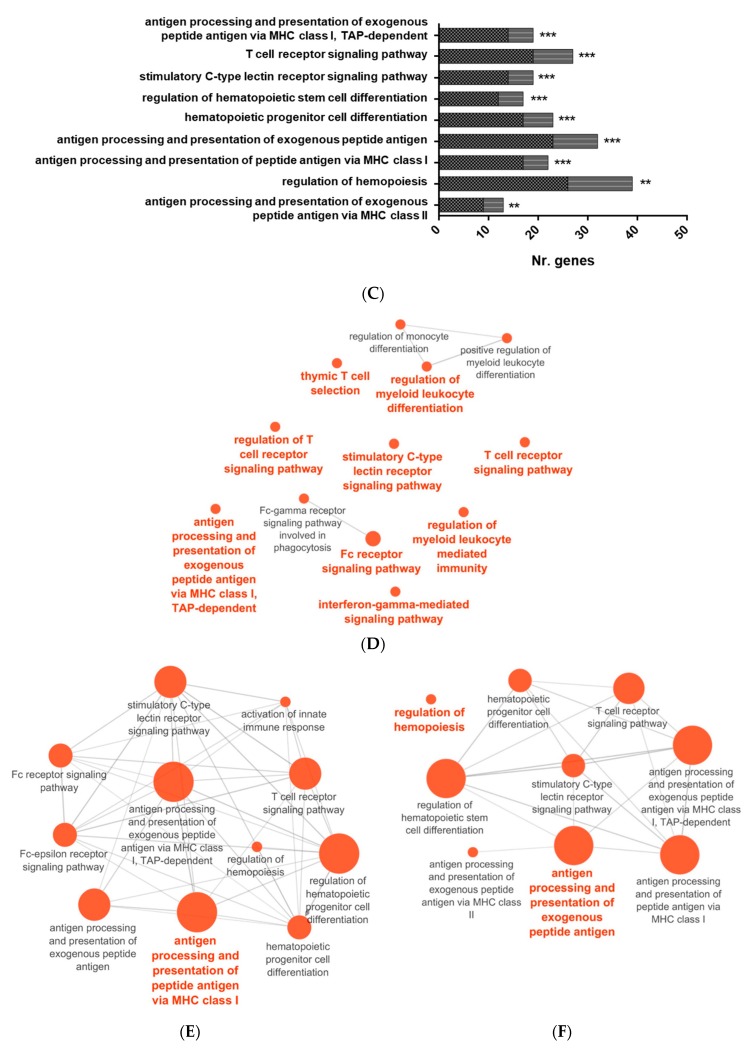
Overrepresented functional pathways in RBCs from GVHSV-immunized fish. Overrepresented GO Immune System Process terms were identified by the Cytoscape ClueGO. (**A**) Overrepresented pathways in the HK-RBC transcriptome profile. (**B**) Overrepresented pathways in the PB-RBC transcriptome profile. (**C**) Overrepresented pathways in the PB-RBC proteome profile. Black squares represent upregulated genes or proteins, and gray squares represent downregulated genes or proteins identified in each GO term. Asterisks denote GO-term significance: * *p*-value < 0.05, ** *p*-value < 0.01 and *** *p*-value < 0.001. Overrepresented terms in the GO Immune System Process network are shown in the (**D**) HK-RBC transcriptome profile, (**E**) PB-RBC transcriptome profile, and (**F**) PB-RBC proteome profile. Each node represents a GO term from an Immune System Process. Node size shows GO term significance (*p*-value); a smaller *p*-value is represented by a larger node size. Edges between nodes indicate the presence of common genes; a thicker line implies a larger overlap. The most significant GO term for each group is labeled.

**Figure 4:**
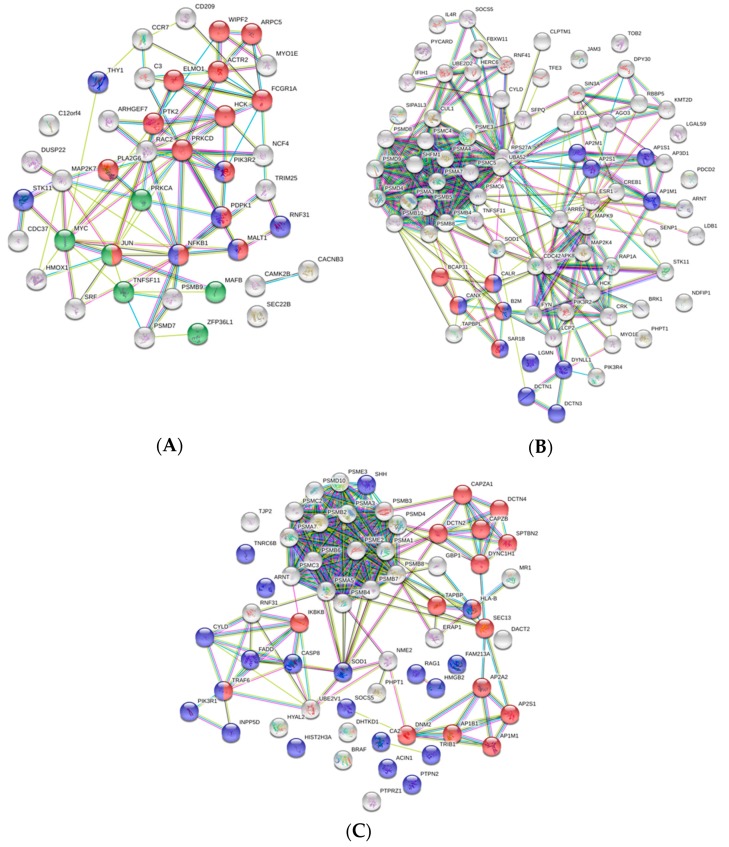
The protein–protein interaction (PPI) network of the set of DEGs/DEPs identified in the overrepresented GO Immune System Process terms. PPI networks were constructed using the STRING software. (**A**) The PPI network of identified DEGs from the HK-RBC transcriptome profile. (**B**) The PPI network of identified DEGs from the PB-RBC transcriptome profile. (**C**) The PPI network of identified DEPs from the PB-RBC proteome profile. Nodes represent proteins, while edges denote the interactions between 2 proteins. Different line colors represent the evidence types used in predicting the associations: gene fusion (red), gene neighborhood (green), gene co-occurrence (blue), co-expression (black), from curated databases (teal), experimentally text-mining (yellow), determined (purple), or protein homology (lilac). The PPI enrichment *p*-value was < 1.0^−16^ for the 3 networks represented. Red nodes denote proteins implicated in (**A**) the Fc receptor signaling pathway (GO:0038093), (**B**) antigen processing and presentation of peptide antigen via MHC class I (GO:0002474), and (**C**) antigen processing and presentation of exogenous peptide antigen (GO:0002478). Blue nodes denote proteins implicated in (**A**) T cell receptor signaling pathway (GO:0050852), (**B**) antigen processing and presentation of exogenous peptide antigen (GO:0002478), and (**C**) regulation of hemopoiesis (GO:1903706). Green nodes denote proteins implicated in the regulation of myeloid leukocyte differentiation (GO:0002761).

Functional pathway enrichment analysis of DEGs in HK-RBCs from GVHSV-immunized individuals using the GO Immune System Process Database revealed the overrepresentation of the following categories: antigen processing and presentation of exogenous peptide antigen via MHC class I, TAP-dependent; regulation of T cell receptor signaling pathway; thymic T cell selection; regulation of myeloid leukocyte differentiation; T cell receptor signaling pathway; stimulatory C-type lectin receptor signaling pathway; regulation of myeloid leukocyte mediated immunity; Fc receptor signaling pathway; and interferon-gamma-mediated signaling pathway (Figure 3A,D) (Supplementary Table S4). Among the DEGs overexpressed in GVHSV-immunized individuals, we were particularly interested in the Fc fragment of IgG receptor Ia (*fcgr1a*) (log_2_ FC = 10.79) and hematopoietic cell kinase (*hck*) (log_2_ FC = 5.97), which are implicated in the Fc receptor signaling pathway; TNF superfamily member 11 (*tnfsf11*) (log_2_ FC = 8.43), which is involved in the regulation of myeloid leukocyte differentiation; and C-C motif chemokine receptor 7 (*ccr7*) (log_2_ FC = 9.67), which is involved in the T cell receptor signaling pathway (Supplementary Table S5). Moreover, the PPI network analysis of DEGs from overrepresented pathways in HK-RBCs from GVHSV-immunized individuals using the GO Immune System Process Database demonstrated a high interaction between the identified genes and corroborated the functional pathway enrichment analysis results (Figure 4A).

### 3.2. RNA Sequencing of PB-RBCs from GVHSV-Immunized Rainbow Trout

The transcriptome profile of FACS single-cell sorted PB-RBCs from GVHSV-immunized individuals (Figure 1) identified 1018 DEGs (FDR < 0.05); 892 were upregulated and 126 were downregulated when compared to PB-RBCs from TFP1-injected individuals (Supplementary Table S6). *gvhsv* gene transcripts were detected, but not significantly (FDR > 0.05), in PB-RBCs from GVHSV-immunized individuals.

Functional pathway enrichment analysis of PB-RBCs from GVHSV-immunized individuals using the GO Biological Process Database revealed the overrepresentation of the following processes: cellular macromolecule metabolic process, cellular nitrogen compound metabolic process, negative regulation of metabolic process, protein localization, response to organic substance, intracellular signal transduction, cellular catabolic process, cellular response to stress, regulation of cell death, hematopoietic or lymphoid organ development, apoptotic signaling pathway, autophagy, and cell surface receptor signaling pathway involved in cell-cell signaling (Figure 2B) (Supplementary Table S7). Among the genes identified, we were particularly interested in the WD repeat domain, phosphoinositide interacting 1 (*wipi1*) (log_2_ FC = 6.69), GABA type A receptor-associated protein (*gabarap*) (log_2_ FC = 5.12), and unc-51 like autophagy activating kinase 1 (*ulk1*) (log_2_ FC = 6.63), which are involved in the autophagy pathway; BCL2-like 1 (*bcl2l1*) (log_2_ FC = 3.93), BCL2-associated athanogene 3 (*bag3*) (log_2_ FC = 5.97), BCL2-associated athanogene 5 (*bag5*) (log_2_ FC = 5.25), BCL2-interacting protein 3 (*bnip3*) (log_2_ FC = 4.78), superoxide dismutase 1 (*sod1*) (log_2_ FC = 5.37), and superoxide dismutase 2 (*sod2*) (log_2_ FC = 6.22), which are involved in the apoptosis signaling pathway and, specifically, the negative regulation of apoptosis and the antioxidant response; TNF superfamily member 11 (*tnfsf11*) (log_2_ FC = 7.16), cytokine receptor-like factor 1 (*crlf1*) (log_2_ FC = 7.08), suppressor of cytokine signaling 3 (*socs3*) (log_2_ FC = 6.95), lymphocyte cytosolic protein 1 (*lcp1*) (log_2_ FC = 5.58), TNF receptor-associated protein 1 (*trap1*) (log_2_ FC = 4.31), and TNF receptor-associated factor 2 (*traf2*) (log_2_ FC = 6.78), which are involved in intracellular signal transduction; and interferon regulatory factor 8 (*irf8*) (log_2_ FC = 6.83) and the suppressor of cytokine signaling 5 (*socs5*) (log_2_ FC = 6.81), which participate in hematopoietic or lymphoid organ development (Supplementary Table S8).

Functional pathway enrichment analysis of PB-RBCs from GVHSV-immunized individuals using the GO Immune System Process Database revealed the upregulation of antigen processing and presentation of peptide antigen via MHC class I (Figure 3B,E) (Supplementary Table S9). Within the DEGs overexpressed in these pathways, we were particularly interested in the following genes: beta-2-microglobulin (*b2m*) (log_2_ FC = 7.37), calnexin (*canx*) (log_2_ FC = 4.42), TAP binding protein-like (*tapbpl*) (log_2_ FC =13.99), and genes related to the proteasome, such as proteasome subunit alpha 3 (*psma3*) (log_2_ FC = 7.17) and proteasome subunit alpha 7 (*psma7*) (log_2_ FC = 6.28) (Supplementary Table S10). Moreover, the PPI network of DEGs from overrepresented pathways in PB-RBCs from GVHSV-immunized individuals in the GO Immune System Process Database demonstrated high interaction between the identified genes (Figure 4B).

### 3.3. Proteome Sequencing of PB-RBC from GVHSV-Immunized Rainbow Trout

The proteome profile of Ficoll-purified PB-RBCs from GVHSV-immunized individuals identified 848 DEPs (FDR < 0.05); 573 proteins were upregulated and 275 proteins were downregulated compared to PB-RBCs from TFP1-injected individuals (Supplementary Table S11). The GVHSV protein was not detected in PB-RBCs from GVHSV-immunized individuals.

Functional pathway enrichment analysis of PB-RBCs from GVHSV-immunized individuals using the GO Biological Process Database revealed the overrepresentation of the following processes: organonitrogen compound metabolic process, cellular nitrogen compound metabolic process, phosphorus metabolic process, negative regulation of macromolecule metabolic process, intracellular transport, regulation of multicellular organismal process, regulation of cellular component organization, regulation of response to stress, nucleobase-containing small molecule metabolic process, cellular component morphogenesis, and mitotic cell cycle process (Figure 2C) (Supplementary Table S12). Within the category of regulation of response to stress and intracellular pathways, we detected overexpression of several nucleoporins, such as nucleoporin 107 (NUP107) (log_2_ FC = 5.44), nucleoporin 155 (NUP155) (log_2_ FC = 3.64), nucleoporin 43 (NUP43) (log_2_ FC = 1.65), nucleoporin 133 (NUP133) (log_2_ FC = 1.72), nucleoporin 85 (NUP85) (log_2_ FC = 4.00), and nucleoporin 88 (NUP88) (log_2_ FC = 3.34). We found particularly interesting the identification of NLR family CARD domain-containing 3 (NLRC3) (log_2_ FC = 3.77), which is involved in the regulation of cellular component organization and in the regulation of response to stress (Supplementary Table S13).

Functional pathway enrichment analysis of PB-RBCs from GVHSV-immunized individuals using the GO Immune System Process Database revealed the overrepresentation of the following pathways: antigen processing and presentation of exogenous peptide antigen via MHC class I (MHCI), TAP-dependent, or via MHC class II (MHCII) and regulation of hematopoiesis (Figure 3C,F) (Supplementary Table S14). Within the pathways related to the antigen presentation, we found particularly interesting the presence of proteins such as major histocompatibility complex, class I, B (HLA-B) (log_2_ FC = 1.76) and TAP binding protein (TAPBP) (log_2_ FC = 2.11) for antigen processing and presentation of exogenous peptide antigen via MHCI, TAP-dependent, as well as dynamin 2 (DNM2) (log_2_ FC = 1.59), dynein cytoplasmic 1 heavy chain 1 (DYNC1H1) (log_2_ FC = 3.48), and SEC13 homolog, nuclear pore and COPII coat complex component (SEC13) (log_2_ FC = 2.85) for antigen processing and presentation of exogenous peptide antigen via MHCII (Supplementary Table S15). Furthermore, we highlight certain proteins overexpressed in PB-RBCs from GVHSV-immunized individuals, such as major histocompatibility complex, class I-related (MR1) (log_2_ FC = 4.98), interleukin 12 receptor subunit beta 2 (IL12RB2) (log_2_ FC = 3.56), tripartite motif-containing 25 (TRIM25) (log_2_ FC = 3.58), tripartite motif-containing 35 (TRIM35) (log_2_ FC = 2.65), interferon-induced protein 35 (IFI35) (log_2_ FC = 2.05), interferon-induced protein 44-like (IFI44L) (log_2_ FC = 3.71), and novel immune-type receptor 9 *nitr9* (log_2_ FC = 4.09) (Supplementary Table S11). The PPI network of DEPs from overrepresented pathways in PB-RBCs from GVHSV-immunized individuals in the GO Immune System Process Database demonstrated high interaction between the identified genes (Figure 4C).

### 3.4. Overrepresented pathways RT-qPCR analysis

**Figure 5:**
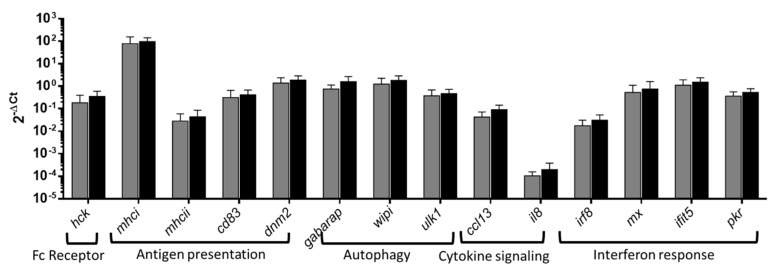
The RT-qPCR analysis of the overrepresented pathways. PB-RBCs were purified from rainbow trout immunized with GVHSV (black bars) or TFP1 (gray bars) at 14 dpi. Gene expression was evaluated by RT-qPCR. Data are displayed as mean ± standard deviation (SD) (*n* = 6). The *ef1α* gene was used as an endogenous control. The Mann–Whitney test was performed to compare PB-RBCs between GVHSV- and TFP1-injected individuals.

Overrepresented pathways in PB-RBCs and HK-RBCs from GVHSV-immunized individuals were validated via RT-qPCR of Ficoll-purified PB-RBCs at 14 dpi. For the Fc receptor signaling pathway (overrepresented in the transcriptome profile of HK-RBC), we measured the *hck* gene expression level, which was upregulated, although without statistical significance (Figure 5). For the antigen presentation pathways (which were overrepresented in the transcriptome analysis of PB-RBC and HK-RBC and in the proteome analysis of PB-RBCs), major histocompatibility complex I (*mhcI*), major histocompatibility complex II (*mhcII*), *dnm2*, and cluster of differentiation 83 (*cd83*) genes were upregulated, although without statistical significance (Figure 5). For the autophagy pathway, the *gabarap*, *ulk1*, and *wipi1* genes had increased expression, but without statistical significance (Figure 5). For the cytokine signaling pathway, the *ccl13* and C-X-C motif chemokine ligand 8 (*il8*) genes were upregulated, but without statistical significance. For the interferon response pathway, interferon regulatory factor 8 (*irf8*), interferon-induced protein with tetratricopeptide repeats 5 (*ifit5*), dsRNA-activated protein kinase R (*pkr*), and interferon-inducible Mx (*mx*) gene expression levels were upregulated, but again without statistical significance (Figure 5). Separately, *gvhsv* gene transcripts were hardly detected (over 35 of 40 Cts) in PB-RBCs from GVHSV immunized individuals.

### 3.5. Leukocyte Proliferation

**Figure 6:**
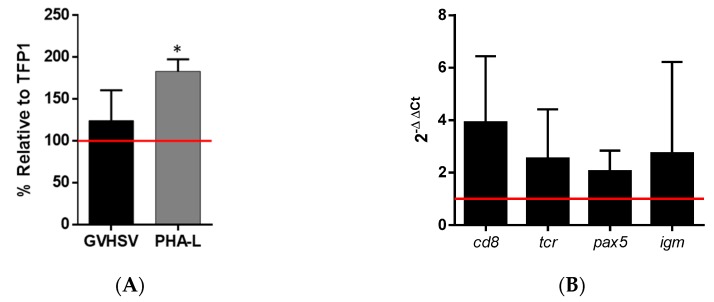
The white blood cell (WBC) proliferation after coculture with GVHSV-transfected RBCs. RBCs and WBCs were purified from the peripheral blood of rainbow trout. WBCs were cocultured with autologous TFP1-transfected RBCs (control), RBCs and PHA-L (positive control), or GVHSV-transfected RBCs. (**A**) WBC proliferation was measured after 7 days as a percentage of fluorescent nuclei (Hoechst stain) using the IN Cell Analyzer and calculated using the following formula: ((number of cell nuclei in WBCs and treated RBCs − number of cell nuclei in untreated RBCs) ÷ (the number of cell nuclei in WBCs and control RBCs – number of cell nuclei in untreated RBCs)) × 100. Data are displayed as mean ± SD (*n* = 4). Data are shown relative to the TFP1 condition (control, red line). A Kruskal–Wallis with Dunn’s multiple comparisons test was performed between each condition and the control. * *p*-value < 0.05). (**B**) WBC gene expression of lymphocyte cell markers was measured at 7 days in cocultures of WBCs and GVHSV- or TFP1-transfected RBCs. Gene expression was evaluated by RT-qPCR. Data are displayed as mean ± SD (*n* = 4). The *ef1α* gene was used as an endogenous control. Data shown are relative to the TFP1 condition (control, red line). A Wilcoxon test was performed to compare coculture of WBCs and GVHSV-transfected RBCs with WBCs and TFP1-transfected RBCs.

GVHSV-transfected RBCs cocultured with autologous WBCs from peripheral blood resulted in the proliferation of WBCs compared to WBCs cocultured with TFP1-transfected RBCs, as observed by the enumeration of cell nuclei (Figure 6A). As a positive control, the coculture of WBCs with untransfected RBCs and stimulation with PHA-L (a well-known lymphocyte mitogen) resulted in a greater proliferation of WBCs compared to other conditions (Figure 6A).

The expression of certain genes related to T cells (cluster of differentiation 8 (*cd8*) and T-cell receptor (*tcr*)) and B cells (paired box gene 5 (*pax5*) and IgM membrane (*igm*)) was upregulated in WBCs cocultured with GVHSV-transfected RBCs compared to WBCs cocultured with TFP1-transfected RBCs (Figure 6B).

### 3.6. Antibody Detection in GVHSV-RBCs Reinfusion/Immunization

**Figure 7:**
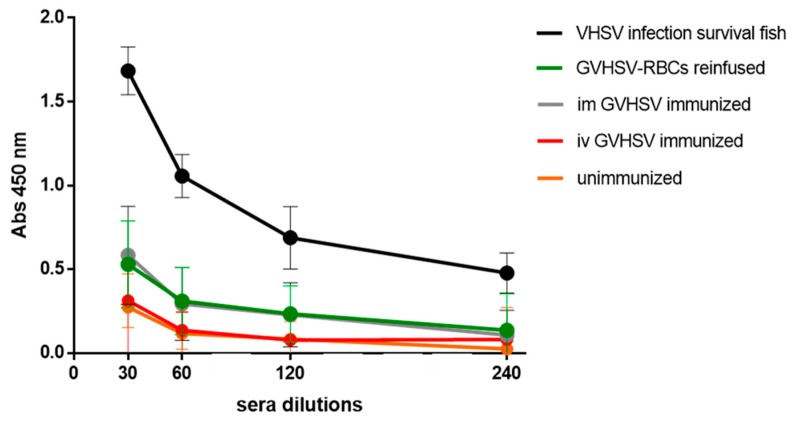
The VHSV-specific antibody detection in serum of GVHSV-transfected RBCs-reinfused/immunized fish. Serum dilution from: (i) im GVHSV-immunized individuals, (ii) iv GVHSV-immunized individuals, and (iii) iv GVHSV-transfected RBCs-reinfused/immunized individuals. Serum obtained from VHSV-challenged survivor trout was used as a positive control, and serum from unimmunized, uninfected fish was used as a negative control. Anti-VHSV antibodies were detected by absorbance readings at 450 nm. Results are expressed as mean ± SD of absorbance (*n* = 4), performed in triplicate.

VHSV-specific IgM was detected in the serum of individuals iv reinfused/immunized with autologous RBCs transfected in vitro with GVHSV at 30 dpi (Figure 7), reaching the same level of antibodies as individuals im immunized with GVHSV DNA vaccine (Figure 7). Anti-VHSV antibodies were not detected in individuals iv immunized with GVHSV DNA vaccine (Figure 7), which resulted in the same levels of absorbance as the negative control.

## 4. Discussion

Currently, DNA vaccination is one of the most effective approaches to prevent viral diseases in aquaculture [2]. DNA vaccines encoding the glycoprotein G gene have demonstrated to be highly effective against fish rhabdoviruses [5,6]. In this study, we determined the role of RBCs in the context of GVHSV DNA vaccination and propose RBCs as mediators of the immune response triggered by the GVHSV DNA vaccine.

Fish RBCs are nucleated cells, and as such, they are able to respond at transcript and protein levels to a stimulus. Further, fish RBCs have been implicated in the immune response against different viruses [9,10,11,12,13,51]. Recently, our laboratory found that rainbow trout RBCs are able to carry a DNA vaccine and respond to the encoded antigen in vitro [14]. Transcriptome profiling of GVHSV-expressing RBCs revealed gene expression changes related to the G-protein coupled receptor (GPCR)-downstream signaling, complement activation, and RAR-related orphan receptor α (RORA) [14]. On the other hand, proteomic profile functional network analysis of GVHSV-transfected RBCs revealed the overexpression of proteins involved in the interferon-stimulated gene 15 (ISG15) antiviral mechanisms, detoxification of reactive oxygen species, antigen presentation of exogenous peptides, and the proteasome [14].

In the present work, the role of RBCs from blood and head kidney tissues of immunized fish was investigated through transcriptomic and proteomic analyses. Rainbow trout head kidney is the major hematopoietic organ in fish [52] and is the location in which phagocytosis, antigen processing, and B cell maturation and differentiation occur [53]. In this regard, we aimed to determine the role of RBCs within the head kidney WBCs, where the main innate and adaptive immune responses to DNA vaccination occur.

Transcriptomic sequencing of FACS single-cell sorted HK-RBCs from GVHSV-immunized fish revealed the overrepresentation of pathways related to the cellular response to chemical stimulus and stress using the GO Biological Process Database. These pathways were also overrepresented in the transcriptome and proteome profile of PB-RBCs. Genes related to responses to cellular stress have been also reported to be modulated in RBCs from the blood of Piscine orthoreovirus (PRV)-challenged Atlantic salmon [13]. In the transcriptome profile of HK-RBCs within these pathways, we highlighted the overexpression of the genes *cxcr4*, *ccl13*, *ccl25*, and the CCL25 receptor *ccr9*, which are all involved in mammalian leukocyte chemotaxis [54,55,56]. The presence of *ccl25/ccr9* in the mammalian intestine has been widely discussed, particularly the involvement of these genes in the development and trafficking of T cells [57]. In teleosts, the presence of *ccl25/ccr9* has been reported mainly in the gut, but also in hematopoietic tissue such as the thymus, spleen, or head kidney [58,59]. The CCL25/CCR9 system has been described as highly conserved throughout vertebrates and recruits homing T cells after oral vaccination in fish [58]. In addition, the upregulation of *ccl25/ccr9* was found after parasitic infection of fish [59]. The role of the CCL25/CCR9 system in the RBC immune response has not been investigated yet and represents an open field of study. On the other hand, it has been reported that RBCs from peripheral blood of PRV-challenged individuals down-regulated the expression of *cxcr4b* and *ccl13* genes [13], in contrast to what we observed in HK-RBCs from GVHSV-immunized individuals.

Functional pathway analysis of the HK-RBC transcriptome profile from GVHSV-immunized fish using the GO Immune System Process Database revealed the overrepresentation of the antigen processing and presentation of exogenous peptide antigen via the MHCI, TAP-dependent pathway. This pathway was also overrepresented in the transcriptomic and proteomic PB-RBC profile from GVHSV-immunized fish. Commonly, MHCI is characterized by endogenous antigen presentation from the degradation of intracellular pathogens and presentation to CD8^+^ T lymphocytes for their clearance [60]. MHCI plays an important role in the defense against viruses [61]. Transcriptomic analysis of PRV-infected RBCs [13] revealed the upregulation of genes related to antigen presentation via MHCI. Additionally, proteomic analysis of rock bream iridovirus (RBIV)-infected RBCs [51] revealed upregulation of antigen processing and presentation of exogenous peptide antigen via MHCI, TAP-dependent pathway. This process suggests the presentation of exogenous peptides in MHCI molecules which is known as cross-presentation [62,63,64,65]. In this process, exogenous peptides are presented on the cell surface together with MHCI molecules through transport via the TAP pathway through the cytosol [63,64,65,66,67,68]. Recently, proteomic profiling of in vitro GVHSV-transfected rainbow trout RBCs revealed the upregulation of antigen processing and presentation of exogenous peptide antigen via the MHCI, TAP-dependent pathway [14]. Antigen presentation of exogenous peptide antigen via MHCI was one the main pathways overrepresented in HK-RBCs and PB-RBCs; overexpression was confirmed at both the transcriptomic and proteomic levels. The presentation of exogenous peptide antigen via MHCI has been especially described for professional APCs [69,70].

Antigen presentation of exogenous peptide via MHCII was also overrepresented in the functional pathway analysis of the PB-RBC proteome from GVHSV-immunized fish. Genes and proteins related to the proteosomal cleavage of exogenous antigen and antigen presentation of exogenous peptides have been reported to be upregulated in GVHSV-transfected RBCs, indicating that RBCs could have the capacity to present DNA vaccine antigens via MHCI or MHCII [14]. Additionally, MHCII gene and protein expression in nucleated RBCs have been recently reported [14,22,29,71]. Currently, MHCII is undergoing functional evaluation in these cells.

Another remarkable pathway overrepresented in the HK-RBC transcriptomic profile was the Fc receptor signaling pathway. The molecular signaling triggered by the union of the immunoglobulin Fc regions with Fc receptors mediates cellular responses that are fundamental in the immune response [72]. HCK, which was upregulated in HK-RBCs from GVHSV-immunized individuals, is a member of the Src family of tyrosine kinases. This family plays an important role in the regulation of innate immune responses [73]. Scr family tyrosine-protein kinases of hematopoietic origin have been suggested to be potential transducers in the activation of monocytes/macrophages [74], participants in the regulation of myeloid cell migration [75], and players in neutrophil activation and recruitment [76]. In contrast, RBCs from PRV-challenged Atlantic salmon have been reported to downregulate *hck* gene expression [13].

Lymphocyte signaling is an important issue to consider in DNA vaccination strategies to improve efficacy [20]. Pathways such as the regulation of myeloid leukocyte differentiation, T cell receptor signaling, regulation of myeloid leukocyte-mediated immunity, and the thymic T cell selection pathways were overrepresented in the HK-RBC transcriptomic profiling, suggesting crosstalk between RBCs and WBCs in the fish head kidney. The T cell receptor signaling pathway was also overrepresented in both the transcriptome and proteome of PB-RBCs. Mammalian RBCs may be inducers of T cell proliferation and contribute to the immune system through crosstalk with leukocytes [77]. Crosstalk between rainbow trout RBCs and other cell types has been also reported. VHSV-exposed RBCs cocultured with TSS, a stromal cell line from rainbow trout spleen, resulted in the upregulation of IFN in both cell types [9]. IFN crosstalk was also observed in RBCs cocultured with the conditioned medium from the rainbow trout gonad-2 (RTG-2) cell line previously exposed to VHSV [9]. GVHSV-transfected RBCs in vitro induced *ifn1* and *mx* gene expression and protected against VHSV infection in RTG-2 cells, in addition to inducing differentiation markers in the rainbow trout monocyte/macrophage-like cell line RTS11 [14]. The present study showed that GVHSV-transfected RBCs induced WBC proliferation in vitro, suggesting that RBCs can stimulate T cells and B cells. However, the role of nucleated RBCs in antigen presentation and crosstalk with WBCs requires additional study.

Functional pathway analysis using the GO Biological Process Database revealed overrepresentation of the autophagy pathway in the PB-RBC transcriptome from GVHSV-immunized fish. Autophagy is a natural, conserved, and self-digestive catabolic process that can be critical for cell survival under stressful conditions, such as viral infection [78,79]. In fish, autophagy has been implicated in viral infections either facilitating [80,81] or inhibiting virus replication [82]. Recently, autophagy has been described in nucleated RBCs as a mechanism for defense against viruses [22,83], and the GVHSV protein is known to be involved in autophagy following immunization with the DNA vaccine [24]. In the present study, we identified a correlation between GVHSV DNA vaccination and autophagy in rainbow trout RBCs. The apoptotic signaling pathway, specifically the negative regulation of apoptosis, was overrepresented in PB-RBCs from GVHSV-immunized individuals. Apoptosis and autophagy play critical roles in maintaining cell homeostasis and are involved in immune system regulation [84]. Overrepresentation of the apoptosis pathway was also detected in the proteomic profile of rock bream RBCs after RBIV infection [51], and apoptosis has been described for RBCs under oxidative stress [85]. On the other hand, an antioxidant response has been reported in vitro in VHSV-infected RBCs and GVHSV-transfected RBCs to likely counteract the oxidative stress triggered by the virus and DNA vaccine [9,14].

The reinfusion/immunization of fish with RBCs transfected in vitro with the GVHSV DNA vaccine revealed the presence of specific antibodies against VHSV in the serum, reaching the same levels of specific antibodies induced by the conventional intramuscular GVHSV DNA vaccination. The idea of RBCs as vaccine carriers has been previously explored in non-nucleated RBCs [15,16,17,18], and RBCs have demonstrated their capacity to induce a humoral response [15]. We have previously demonstrated that rainbow trout nucleated RBCs can respond to and express GVHSV DNA vaccine in vitro. In this study, we demonstrated that RBCs can mount an innate immune response in response to a DNA vaccine in vivo, and moreover, they can induce a humoral immune response.

The use of cytokine genes as vaccine adjuvants has been shown to improve IgM titer, lymphocyte proliferation, and virus protection in glycoprotein G DNA vaccination of rainbow trout [86]. The use of type I interferon as a DNA vaccine adjuvant has also been shown to improve protection against the virus, augmentation of antibody response, and migration of B and CD8^+^T cells [87]. Nucleated RBCs are able to upregulate interferon and interferon-inducible genes and proteins [8,9,10,11,12,13,14]. This link between the innate and adaptive immune responses triggered by RBCs implicates these cells as potential targets for DNA vaccination. Moreover, the involvement of HK-RBCs and PB-RBCs from GVHSV-immunized individuals in the antigen presentation of exogenous peptide antigen via MHCI, as well as the capacity of PB-RBCs to induce WBC proliferation and the ability of GVHSV-transfected PB-RBCs to induce a humoral immune response, lead us to suggest that RBCs may behave as APC-like cells.

The concept of atypical or no professional APCs has been previously explored in mammals. Some cells, such as mast cells, basophils, eosinophils, innate lymphoid cells [88], and neutrophils [89], have been classified as atypical APCs. According to Kambayashi and Laufer [88], atypical APCs differ from professional APCs (dendritic cells, B cells, and macrophages) in their non-constitutive expression of MHCII molecules and the incapacity (or unknown capacity) to prime naïve CD4^+^T cells [88]. Studies in neutrophils revealed that these cells can express MHCII and costimulatory molecules under activated/stimulatory conditions, present antigen to CD4^+^T cells, crosstalk with other leukocyte populations, respond by synthesizing cytokines, and link the innate and adaptive immune response, among other functions [89]. Nucleated RBCs share several of these qualities with neutrophils. Rainbow trout RBCs can upregulate MHCII under stimulatory conditions, such as GVHSV transfection [14]. In the present study, we detected the mRNA expression of MHCII and the overrepresentation of antigen processing and presentation of exogenous peptide via MHC II process at a proteomic level in PB-RBCs from GVHSV-immunized individuals. Additionally, RBCs can engage in crosstalk with other cell types by releasing cytokines under a stimulus in vivo, as we show in this manuscript, and in vitro, as previously described [9,14]. Thus, RBCs may participate in part of the humoral response as DNA vaccine carriers. The findings described here have led us to suggest nucleated RBCs as potential atypical APCs. Cassatella and colleagues compared neutrophils (atypical APCs) with professional APCs and suggested that the high number of atypical APCs found in the immunization site could compensate for their lower capacity for antigen presentation compared with professional APCs [89]. As such, and considering the high number of RBCs present in an organism and their participation in the innate and adaptive immune responses triggered by DNA immunization in vivo, nucleated RBCs may be ideal target cells for adjuvant/vaccination strategies.

## 5. Conclusions

The transcriptomic and proteomic prolife of RBCs after im DNA immunization of rainbow trout revealed the overrepresentation of immune system-related processes, such as the presentation of exogenous peptides. It is noteworthy to highlight that some of the immune response mechanisms that we found overrepresented in the present work have been previously identified in rainbow trout RBCs transfected with GVHSV in vitro. In addition, apart from corroborating previous results, this is the first time that these processes are found in vivo, after im DNA immunization, a scenario where RBCs are not the direct target of the DNA vaccine. This results led us to suggest that RBCs could act as mediator cells of the immune processes triggered by DNA immunization. Moreover, RBCs carrying a DNA vaccine were able to induce a humoral response in fish and stimulate proliferation in leukocytes. All of this would lead us to suggest the RBCs of fish as APC-like cells.

## Figures and Tables

**Table 1:** The list of primers and probes used.

Gene	Forward Primer	Reverse Primer	Probe	Reference or Accession Number
*ccl13*	CCTCTTCAACAAGTGGTTTCTCTCA	AGAAGGGTCAACACAAAATGTCTTC	-	NM_001160689.1
*cd8*	GAC TGC TGG CTG TGG CTT CC	CCC CGG AGC TGC CAT TCT	-	[25]
*cd83*	TTGGCTGATGAT TCTTTCGATATC	TGCTGCCAGGAG ACACTTGT	TCCTGCCCAATG TAACGGCTGTTG	[35]
*dnm2*	GTCAACAAGTCCATCAGGGATCT	CAACTCAGAATGGATGAAGTCTTTAGC	-	[14]
*ef1α*	ACCCTCCTCTTGGTCGTTTC	TGATGACACCAACAGCAACA	GCTGTGCGTGACATGAGGCA	[36]
*gabarap*	CCTCATCCATCCATTT TTACCTCTT	ATTCAACCGAAATCCCC ATCT	TCTGAATTTTATTTG CCTCCGGGTCTCC	[22]
*gvhsv*	GGGCCTTCCTTCTACTGGTACTC	CGGAATCCCGTAATTTGGAAT	CTGTTGCTGCAAGGCGTCCCCT	[37]
*hck*	CCATCTCCACTGGCCCTACA	TACCCTCATAGTCATACAGTGCGATAG	-	XM_021567092.1
*ifit5*	CCCTGCCCTCATCTTTCTTCT	CCCTCAATGACTCTGACAAGCA	CCAGCTTCGGCCTGTTTCTGTTCCA	[14]
*igm*	AAAGCCTACAAGAGGGAGACCGAT	AGAGTTATGAGGAAGAGTATGATGAAGGTG	CTCGTGTTGACTGACTGTCCATGCAGCAAC	[38]
*il8*	AGAGACACTGA GATCATTGCCAC	CCCTCTTCATTTG TTGTTGGC	TCCTGGCCCTCC TGACCATTACTG AG	[39,40]
*irf8*	CCGAGGAGGAGCAGAAGAGTAAAAG	GCGGCATTGAAAGAACCCAT	-	[41]
*mhcI*	GACAGTCCGTCCCTCAGTGT	CTGGAAGGTTCCATCATCGT	-	[42]
*mhcII*	TGCCATGCTGATGTGCAG	GTCCCTCAGCCAGGTCACT	CGCCTATGACTTCTACCCCAAACAAAT	[43]
*mx1-3*	TGAAGCCCAGGATGAAATGG	TGGCAGGTCGATGAGTGTGA	ACCTCATCAGCCTAGAGATTGGCTCCCC	[44]
*pax5*	ACGGAGATCGGATGTTCCTCTG	GATGCCGCGCTGTAGTAGTAC	-	[45]
*pkr*	ACACCGCGTACCGATGTG	GGACGAACTGCTGCCTGAAT	CACCACCTCTGAGAGCGACACCACTTC	[9]
*tcr*	AGCACCCAGACTGCCAAGCT	GAGGAGCCCTGGAACTCCA	TCT TCA TCG CTA AGA GTA CCT TCT ATG GCC TGG T	[25]
*ulk1*	CTTCTGCTGCTGGGTCTTCTG	GGTGACGGAAGAACTCCTCAAA	CGAAACCACAAGGACCGCATGGA	[22]
*wipi1*	CAAAGACATGAAGCTG CTGAAGA	GGTTCACAGAGAGGGC ACAGA	CTCAACACGCCCCACAA CCCCT	XM_021581280.1

## Supplementary Materials

The following are available online at https://www.mdpi.com/2076-393X/7/3/60/s1, Table S1: List of DEGs identified in HK-RBCs from GVHSV-immunized individuals, by transcriptomic sequencing; Table S2: GO Biological Process terms identified in HK-RBCs from GVHSV-immunized individuals, by transcriptomic sequencing; Table S3: List of GO Biological Process terms and associated DEGs identified in HK-RBCs from GVHSV-immunized individuals, by transcriptomic sequencing; Table S4: GO Immune System Process terms identified in HK-RBCs from GVHSV-immunized individuals, by transcriptomic sequencing; Table S5: List of GO Immune System Process terms and associated DEGs identified in HK-RBCs from GVHSV-immunized individuals, by transcriptomic sequencing; Table S6: List of DEGs identified in PB-RBCs from GVHSV-immunized individuals, by transcriptomic sequencing; Table S7: GO Biological Process terms identified in PB-RBCs from GVHSV-immunized individuals, by transcriptomic sequencing; Table S8: List of GO Biological Process terms and associated DEGs identified in PB-RBCs from GVHSV-immunized individuals, by transcriptomic sequencing; Table S9: GO Immune System Process terms identified in PB-RBCs from GVHSV-immunized individuals, by transcriptomic sequencing; Table S10: List of GO Immune System Process terms and associated DEGs identified in PB-RBCs from GVHSV-immunized individuals, by transcriptomic sequencing; Table S11: List of DEPs identified in PB-RBCs from GVHSV-immunized individuals, by proteomic sequencing; Table S12: GO Biological Process terms identified in PB-RBCs from GVHSV-immunized individuals, by proteomic sequencing; Table S13: List of GO Biological Process terms and associated DEPs identified in PB-RBCs from GVHSV-immunized individuals, by proteomic sequencing; Table S14: GO Immune System Process terms identified in PB-RBCs from GVHSV-immunized individuals, by proteomic sequencing; Table S15: List of GO Immune System Process terms and associated DEPs identified in PB-RBCs from GVHSV-immunized individuals, by proteomic sequencing; Figure S1: FACS single-cell sorting of HK-RBCs and PB-RBCs. A) Representative dotplot and histogram showing selected population for FACS single-cell sorted HK-RBCs using BD FACSJazz™ cell sorter. B) 10^2^ purified HK-RBCs and C) 10^6^ purified PB-RBCs stained with SYTO RNASelect and purified by FACS using BD FACSJazz™ cell sorter for transcriptome analysis. Brightfield and FITC images were taken at 10× magnification. D) RBCs after Ficoll gradient purification for proteome analysis. The brightfield image was taken at 20× magnification. Images were taken with the IN Cell Analyzer 6000 Cell Imaging system.
