# Supplementary material for: Potential Role of Rainbow Trout Erythrocytes as Mediators in the Immune Response Induced by a DNA Vaccine in Fish
Source: Vaccines (Basel). 2019 Jul 3;7(3):60. doi: 10.3390/vaccines7030060 (PMC6789471; doi:10.3390/vaccines7030060)

Figure S1: FACS single-cell sorting of HK-RBCs and PB-RBCs. A) Representative dotplot and histogram showing selected population for FACS single-cell sorted HK-RBCs using BD FACSJazz™ cell sorter. B) 10^2 purified HK-RBCs and C) 10^6 purified PB-RBCs stained with SYTO RNASelect and purified by FACS using BD FACSJazz™ cell sorter for transcriptome analysis. Brightfield and FITC images were taken at 10× magnification. D) RBCs after Ficoll gradient purification for proteome analysis. The brightfield image was taken at 20× magnification. Images were taken with the IN Cell Analyzer 6000 Cell Imaging system.

A

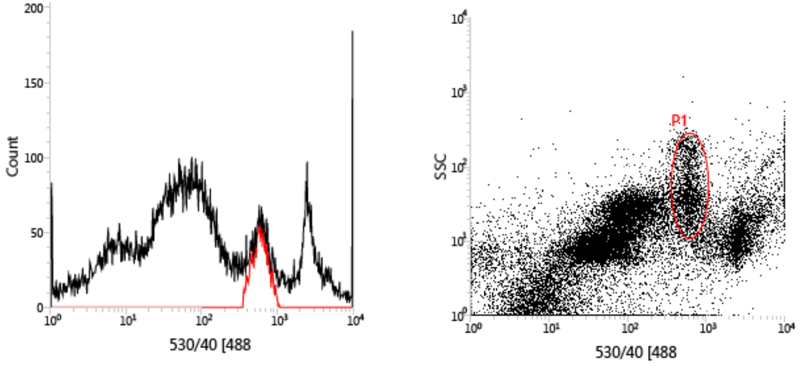

B

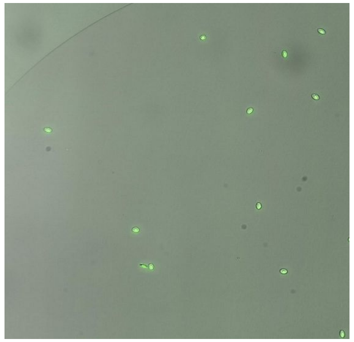

C

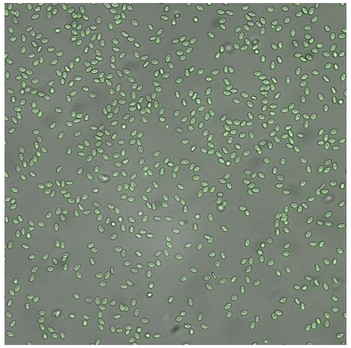

D

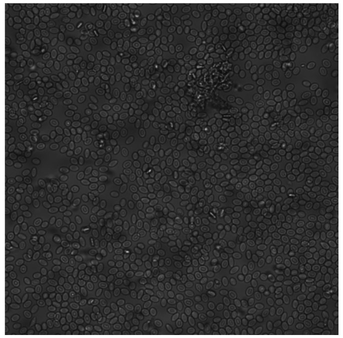

Supplement: Supplementary file 1 [file vaccines-07-00060-s001.zip › Supplementary Material_Puente-Marin_Ortega-Villaizan_Vaccines_2019/Puente-Marin_Ortega-Villaizan_2019_Vaccines_Supplementary Figure S1.pdf]
